# Anti-melanoma Differentiation-Associated Protein 5 (MDA5)-Positive Dermatomyositis With Rapidly Progressive Interstitial Lung Disease (ILD): A Rare and Lethal Entity to Recognize Early

**DOI:** 10.7759/cureus.75864

**Published:** 2024-12-17

**Authors:** Rita Bernardino, Inês Ferreira, André Valente, Conceição Loureiro

**Affiliations:** 1 Internal Medicine, Hospital Curry Cabral - Centro Hospitalar Universitário Lisboa Central, Lisbon, PRT; 2 Internal Medicine, Centro Hospitalar de Lisboa Central, Lisbon, PRT; 3 Internal Medicine, Unidade Local de Saúde de São José, Lisbon, PRT

**Keywords:** anti-mda5 amyopathic dermatomyositis, autoimmune myopathy, immunosuppressive therapy, infectious lung disease, organizing pneumonia, rapidly progressive interstitial lung disease

## Abstract

Anti-melanoma differentiation-associated protein 5 (anti-MDA5) clinically linked amyopathic dermatomyositis (CADM) is a rare autoimmune condition strongly linked to rapidly progressive interstitial lung disease (RP-ILD), a life-threatening complication. We present a 63-year-old female patient with anti-MDA5-positive CADM, who developed RP-ILD with an imaging pattern consistent with organizing pneumonia. She presented with Gottron’s papules, periungual erythema, progressive dyspnea, and anorexia. Despite timely initiation of combination immunosuppressive therapy with corticosteroids, rituximab, and mycophenolate, her disease progressed rapidly, complicated by infections and treatment intolerance, culminating in acute respiratory failure.

This case underscores the aggressive nature of anti-MDA5-positive CADM with RP-ILD, highlighting the critical need for prompt recognition and comprehensive multidisciplinary care. Urgent research efforts are essential to develop and refine treatment strategies for this life-threatening disease.

## Introduction

Dermatomyositis (DM) is a rare idiopathic inflammatory myopathy that primarily affects the skin and muscles, with hallmark cutaneous features such as Gottron’s papules and heliotrope rash and systemic involvement, including interstitial lung disease (ILD) [1]. A specific subtype known as clinically amyopathic dermatomyositis (CADM) is characterized by minimal or absent muscle weakness, while extramuscular involvement, particularly ILD, is often more prominent [2,3]. Within CADM, the presence of anti-melanoma differentiation-associated protein 5 (anti-MDA5) autoantibodies defines a distinct clinical phenotype strongly linked to rapidly progressive interstitial lung disease (RP-ILD), a life-threatening pulmonary complication with high morbidity and mortality [3,4].

Anti-MDA5-positive CADM poses significant diagnostic and therapeutic challenges due to its rapid progression and poor response to treatment. Diagnosis relies on clinical evaluation, the detection of myositis-specific autoantibodies (like anti-MDA5), and imaging findings suggestive of interstitial lung involvement, such as areas of ground-glass opacities (GGOs) or an organizing pneumonia (OP) pattern on computed tomography (CT) scans [5]. Management typically requires a combination of high-dose corticosteroids and other immunosuppressants, such as rituximab, mycophenolate, and cyclophosphamide [6]. However, treatment is often complicated by drug-related adverse effects and opportunistic infections, as profound immunosuppression is required to control the disease. Despite aggressive immunosuppressive therapy, mortality remains high, largely due to the refractory nature of RP-ILD [7].

The epidemiology of anti-MDA5-positive CADM reveals significant geographic differences. The condition is more prevalent in Asian populations, where anti-MDA5 antibodies are found in up to 60% of dermatomyositis cases. In comparison, the prevalence is significantly lower in European populations, where anti-MDA5 antibodies are detected in only 7%-16% of DM cases [8]. This variation highlights the potential influence of genetic and environmental factors on disease susceptibility and presentation [9].

Here, we present the case of a 63-year-old woman with anti-MDA5-positive CADM and RP-ILD. The patient exhibited characteristic cutaneous findings, progressive respiratory symptoms, and significant imaging changes indicative of ILD. Despite the initiation of a comprehensive immunosuppressive regimen, her disease followed a rapidly progressive course with severe pulmonary involvement and treatment-related complications. This case highlights the urgent need for more effective treatment protocols and underscores the complexity of managing this rare, life-threatening condition.

## Case presentation

A 63-year-old Caucasian woman presented with photosensitive, non-pruritic maculopapular lesions on her face and anterior chest wall, which had first appeared approximately five months prior to hospitalization. Over the following weeks, she developed ulcerations on the cervical mucosal surfaces that resolved spontaneously. This was followed by the appearance of erythematous papules on her elbows, along with periungual erythema and telangiectasias. She also reported progressive fatigue and exertional dyspnea, which eventually limited her ability to perform basic activities of daily living. Notably, her fatigue and dyspnea were not associated with symptoms or signs of heart failure. Additionally, she experienced a significant, unintentional weight loss of 10 kg over the course of one month. She did not report any fever, night sweats, nausea, vomiting, dysphagia, reflux symptoms, appetite loss, or changes in bowel habits. Importantly, she had no complaints of muscle weakness. A recent gastrointestinal endoscopy and colonoscopy, performed several years after the removal of benign polyps, revealed no significant abnormalities. Likewise, a mammogram conducted earlier that year and a gynecological evaluation, including a transvaginal ultrasound, were both unremarkable. 

Her medical history was notable for essential hypertension, dyslipidemia (with statin therapy discontinued due to statin-induced myalgias), multinodular goiter with benign cytology, allergic rhinitis, and long-term penicillin prophylaxis for recurrent streptococcal infections. She also had a history of severe allergies, including anaphylaxis to corn derivatives. Her family history was negative for malignancies or autoimmune diseases. She had no history of smoking or significant environmental exposures. Additionally, she was up to date on all recommended vaccinations.

**Figure 1:**
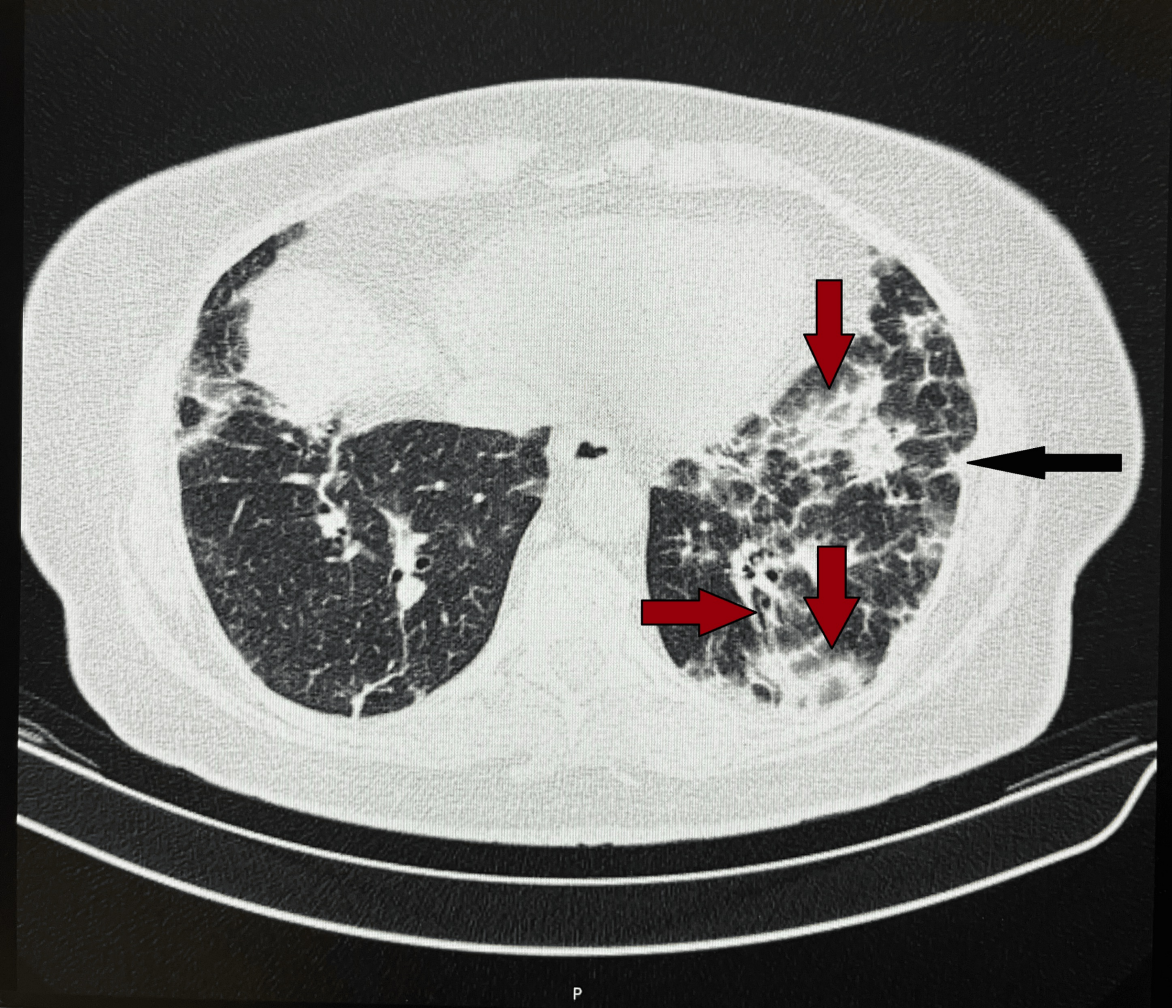
First lung CT scan (axial view) CT: Computed Tomography The black arrow shows subpleural consolidation in the left lung; the red arrows show ground-glass opacities and bronchiectasis in the left lung.

The patient initially presented to her primary care physician with complaints of weight loss, progressive fatigue, exertional dyspnea, and skin changes. These findings prompted the physician to request a CT scan of the lungs for further evaluation. The CT scan revealed multiple subpleural pulmonary nodules ranging from 5 to 11 mm, along with GGOs and subpleural consolidations in the lower lobes. No pleural effusions or masses were observed (Figure 1). Two weeks later, based on these findings and the persistence of her symptoms, she was admitted to the internal medicine ward for further investigation. 

During hospitalization, the patient was noted to have skin alterations suggestive of dermatomyositis, including erythematous papules on the elbows, periungual erythema, and telangiectasias. In response to these findings, a dermatology consultation was requested. Simultaneously, a comprehensive diagnostic workup was initiated to investigate potential infectious, inflammatory, and autoimmune causes, as well as to exclude a potential neoplastic cause, given her significant weight loss. The dermatologist performed a skin biopsy on the patient's face to further evaluate the cutaneous findings. Concurrently, an autoimmune panel was ordered, revealing antinuclear antibody (ANA) positivity (1:160, fine granular) and low-affinity anti-double-stranded DNA (anti-dsDNA) antibodies, along with weakly positive anti-melanoma differentiation-associated gene 5 (anti-MDA5) antibodies (Table 1). An extended autoimmune panel, including antibodies associated with pulmonary involvement, did not yield additional significant findings, and complement levels (C3 and C4) were within normal limits. Treatment for the dermatologic manifestations was initiated with topical corticosteroids and topical tacrolimus 0.1%.

**Table 1:** Key abnormal laboratory findings

Parameter	Result	Reference Range
Erythrocyte Sedimentation Rate (ESR)	53 mm/hr	< 20 mm/hr
Ferritin	791 ng/mL	30 - 400 ng/mL
Anti-Melanoma Differentiation-Associated Gene 5 Antibody (Anti-MDA5)	Weak positive initially; repeated test positive	Negative
Antinuclear Antibody (ANA)	1:160, fine granular	Negative
Anti-Double-Stranded DNA Antibody (Anti-dsDNA)	Low affinity	Negative

Infectious serologies for HIV, hepatitis B, hepatitis C, cytomegalovirus, Epstein-Barr virus, and syphilis were negative. Aldolase, creatine kinase (CK), and myoglobin levels were within normal limits, indicating no significant muscle injury. Serum vitamin B12 and folic acid levels were normal, as were angiotensin-converting enzyme levels. Thyroid function tests were also within normal limits, excluding thyroid dysfunction as a potential cause. NT-proBNP levels were within the normal range, ruling out significant cardiac strain or heart failure. Notably, there was no elevation in C-reactive protein or leukocyte count; however, the erythrocyte sedimentation rate was elevated, and ferritin levels were markedly high, consistent with ongoing inflammation. Renal and liver function tests, coagulation parameters, and serum electrolytes were all within normal limits. No anemia was detected, and total protein levels and serum protein electrophoresis did not reveal any abnormalities.

**Figure 2:**
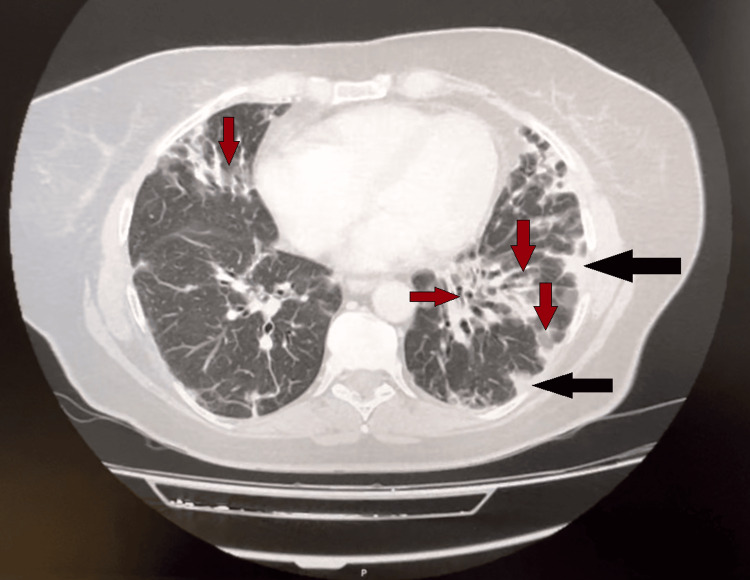
Axial view of the lungs from a CT scan of the chest, abdomen, and pelvis CT: Computed Tomography The black arrows show subpleural consolidations in the left lung, and the red arrows show ground-glass opacities and bronchiectasis in the right and left lungs.

A CT scan of the chest, abdomen, and pelvis was performed to exclude malignancy. The imaging revealed progression of pulmonary findings, including worsening GGOs, new fibrotic changes, and increased subpleural consolidations in both lung bases. Additionally, bilateral hilar and mediastinal lymph node hypertrophy was observed, likely as a reactive response to the ongoing inflammatory process. No significant abnormalities were identified in the abdomen or pelvis (Figure 2). These findings indicated significant parenchymal damage consistent with ILD. Bronchoalveolar lavage (BAL) analysis showed: "Composition of ciliated cylindrical cells, alveolar macrophages, lymphocytes, and a few polymorphonuclear cells. Investigation for neoplastic cells: Negative." These results pointed to an inflammatory etiology. Considering the radiological findings and in consultation with the pulmonology team, OP was deemed the most likely diagnosis. Despite the progression of pulmonary abnormalities, the patient remained clinically stable from a respiratory perspective and did not require supplemental oxygen during her hospital stay.

Given the combination of clinical findings, imaging, and laboratory results, there was strong clinical suspicion for amyopathic dermatomyositis (ADM) with ILD. The results of the skin biopsy, which became available later, confirmed histological features consistent with dermatomyositis. Following this result, anti-MDA5 antibodies were repeated and returned strongly positive, confirming the diagnosis of ADM associated with ILD.

She was subsequently referred to an expert team in autoimmune diseases at another hospital, in collaboration with pulmonology. Pulmonary function tests revealed a restrictive ventilatory defect with reduced forced vital capacity at 60% of predicted and diffusing capacity for carbon monoxide at 40% of predicted. A six-minute walk test demonstrated severe exercise limitation, with a distance of 240 meters (46% of predicted) and exertional hypoxemia, with oxygen saturations persistently below 90% (Table 2). Arterial blood gas analysis on room air and at rest revealed very mild hypocapnia and respiratory alkalosis. 

**Table 2:** Abnormal immunological and pulmonary function findings CD3: Cluster of Differentiation 3; CD4: Cluster of Differentiation 4; CD8: Cluster of Differentiation 8 * Note: The reference value for the 6-Minute Walk Test distance (>500 m) is a general guideline and may not accurately reflect population-specific norms. In Portugal, predicted values vary based on age, sex, and body mass index (BMI), and population-specific reference equations should be used for a more accurate assessment.

Parameter	Result	Reference Range
Forced Vital Capacity (FVC)	60% predicted	> 80% predicted
Diffusing Capacity for CO (DLCO)	40% predicted	> 75% predicted
6-Minute Walk Test (Distance)	240 m	> 500 m *
Oxygen Saturation During Walk	Persistently < 90%	> 90% during exercise
Immunoglobulin E (IgE)	134 UI/mL	< 100 UI/mL
Total T Lymphocytes (CD3)	398 L cells/µL	690 - 2540 cells/µL
T-Helper Lymphocytes (CD3+CD4+)	344 L cells/µL	410 - 1590 cells/µL
T-Suppressor Lymphocytes (CD3+CD8+)	58 L cells/µL	190 - 1140 cells/µL
T-Suppressor Lymphocytes (CD3+CD8+) %	9 L%	13 - 41%
CD4/CD8 Ratio	5.89	1.0 - 2.5

Further blood analyses included immunoglobulin levels and flow cytometry to assess lymphocyte populations. Immunoglobulin testing revealed elevated IgE levels, while IgG, IgA, and IgM levels were within normal limits. Flow cytometry demonstrated a reduction in total T lymphocytes (CD3), reduced T-helper lymphocytes (CD3+CD4+), and a marked reduction in T-suppressor lymphocytes (CD3+CD8+). These findings resulted in an increased CD4/CD8 ratio. Natural killer (NK) cells (CD16+CD56+CD3-) and B lymphocytes (CD19) were within normal limits (Table 2).

These findings revealed impaired pulmonary function, evidenced by the restrictive ventilatory defect, reduced gas exchange capacity, and exertional hypoxemia. Additionally, significant reductions in T-cell subpopulations, particularly CD8+ lymphocytes, along with an elevated CD4/CD8 ratio, suggested underlying immune dysregulation. 

The patient was started on high-dose deflazacort (60 mg/day for three weeks), with a planned taper to 40 mg/day thereafter, along with immunosuppressive therapy with rituximab (1 g, administered twice, 15 days apart). Mycophenolate mofetil was initiated as an additional immunosuppressant. The decision was made to defer cyclophosphamide due to her low CD8 count and history of recurrent infections, which raised concerns about a possible primary immunodeficiency. However, a formal and comprehensive investigation for primary immunodeficiency was not conducted due to the urgency of addressing her rapidly progressing disease. Prior to initiating immunosuppressive therapy, the patient was vaccinated against pneumococcus and influenza to reduce the risk of severe respiratory infections, which are known to significantly impact the clinical course of RP-ILD.

**Figure 3:**
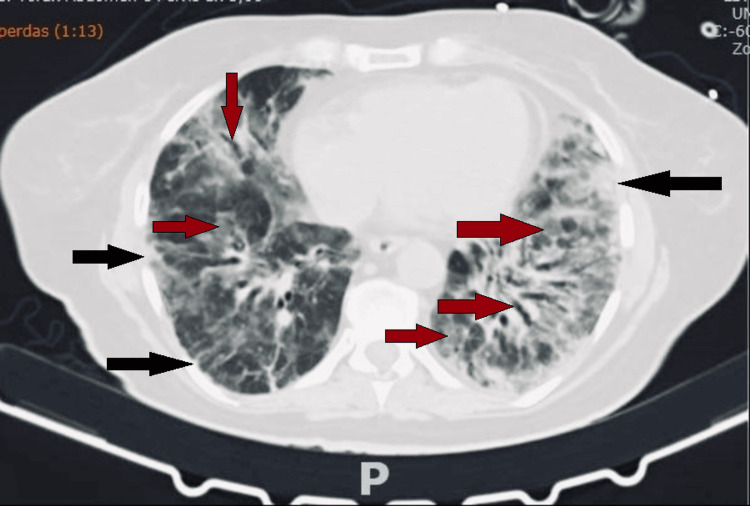
Third lung CT scan (axial view) CT: Computed Tomography The black arrows show subpleural consolidations in the right and left lung; the red arrows show ground-glass opacities and bronchiectasis in the right and left lung.

Approximately three months after the initial presentation, the patient's condition significantly deteriorated, requiring readmission to the hospital for urgent management. She developed laryngitis, followed by persistent fever and worsening respiratory symptoms, accompanied by hypoxemia, with a partial pressure of oxygen (pO₂) of 60 mmHg. During this period, she also experienced a hypersensitivity reaction to mycophenolate, leading to its discontinuation. A CT scan of the lungs revealed extensive new GGOs affecting 50% to 75% of the lung parenchyma, accompanied by traction bronchiectasis and architectural distortion, particularly in the lower lobes. Additionally, there was an increase in subpleural consolidations, the emergence of thick linear subpleural densities, and scattered subpleural micronodules, all of which reflect substantial disease progression and extensive parenchymal damage (Figure 3). Given the obvious clinical deterioration, inflammatory markers such as ferritin, interleukin-6 (IL-6), and anti-MDA5 antibodies were not deemed necessary for disease monitoring, as the severity of her condition was already evident. 

The patient was admitted to the ICU with acute respiratory failure and was initially managed with high-flow oxygen therapy. To address the possibility of a superimposed infection, she was started on broad-spectrum antibiotics, including meropenem, ceftriaxone (CTX), and azithromycin (AZT). High-dose corticosteroids, including pulse-dose methylprednisolone, were also administered as part of her treatment regimen. A repeat BAL was performed, which demonstrated elevated galactomannan levels, raising suspicion of a fungal infection. As a result, treatment with liposomal amphotericin B was initiated. Additionally, she underwent four days of plasmapheresis and received high-dose intravenous immunoglobulin as part of her immunomodulatory treatment strategy.

Despite these intensive therapeutic efforts, her clinical and radiologic status continued to worsen, with progressive hypoxemia and respiratory distress. She was subsequently indicated for invasive mechanical ventilation; however, the patient declined intubation and mechanical ventilation. Her respiratory condition deteriorated further, and she succumbed to respiratory failure shortly thereafter.

## Discussion

CADM is a distinct subtype of idiopathic inflammatory myopathies (IIMs), characterized by classic DM cutaneous features without clinically apparent muscle weakness. When associated with anti-MDA5 antibodies, CADM becomes particularly aggressive, often presenting with RP-ILD, which is a leading cause of morbidity and mortality [8]. This case illustrates the diagnostic and therapeutic challenges posed by this rare disease variant.

The anti-MDA5 antibody is a key biomarker in DM and CADM, with a well-established association with RP-ILD. Its pathophysiological role involves endothelial injury, cytokine storm, and excessive immune activation, all of which contribute to aggressive lung inflammation and fibrosis [10]. In this case, progressive hypoxemia and imaging findings of ground-glass opacities, consolidations, and subpleural fibrosis aligned with the classic radiological features of anti-MDA5+ RP-ILD.

The clinical presentation of CADM poses unique diagnostic challenges. Unlike classic DM, CADM lacks muscle weakness, and its cutaneous signs, such as Gottron’s papules and periungual erythema, may be subtle and easily overlooked. In this case, early recognition of cutaneous findings prompted an autoimmune workup, leading to the identification of anti-MDA5 antibodies and confirmation of DM through skin biopsy. This approach highlights the importance of clinical vigilance and the role of serological and histological evaluation in diagnosing IIMs.

Management of anti-MDA5-positive CADM with RP-ILD requires a multifaceted immunosuppressive approach. First-line therapies typically include high-dose corticosteroids, often in combination with immunosuppressants such as cyclophosphamide, tacrolimus, or mycophenolate mofetil [6]. In this case, rituximab was selected as a key agent due to its efficacy in refractory DM. Given the patient's low CD8 lymphocyte count and history of recurrent infections, cyclophosphamide was not considered a viable option, reflecting the challenge of balancing autoimmune control with infection risk.

The course of treatment was further complicated by severe opportunistic infections, including influenza B and fungal infection, which are frequently seen in immunosuppressed patients. Vaccination against pneumococcus and influenza is crucial before initiating immunosuppressive therapy, and prophylactic treatment for Pneumocystis jirovecii pneumonia should be considered [11]. Despite these measures, the patient’s infection burden significantly affected therapeutic options and clinical outcomes.

As salvage therapies, plasmapheresis and intravenous immunoglobulin have been explored in RP-ILD, with the goal of removing circulating autoantibodies and modulating immune responses. These therapies were employed in this case but failed to halt disease progression. While evidence supports their potential role in refractory cases, success depends on early initiation and the extent of pulmonary fibrosis [6].

Various biomarkers have been identified and are routinely used in clinical practice to predict disease activity and severity. These include anti-MDA5 antibodies, ferritin, and interleukin-6 [12]. While these biomarkers are valuable in guiding clinical decisions, their relevance was diminished in our patient’s case due to the evident clinical deterioration, which provided sufficient indication of disease progression.

The presence of anti-MDA5 antibodies is a well-established marker of poor prognosis, particularly in cases complicated by RP-ILD [13]. Mortality is frequently associated with progressive respiratory failure and secondary infections. This case highlights the urgent need for early diagnosis and prompt therapeutic intervention to improve patient outcomes. The absence of a standardized treatment algorithm for anti-MDA5-positive CADM further emphasizes the ongoing need for targeted therapies and continued research aimed at improving outcomes in this rare and life-threatening disease.

## Conclusions

Anti-MDA5-positive CADM with RP-ILD presents a significant clinical challenge with a high risk of mortality. The absence of muscle weakness often delays diagnosis, and once RP-ILD sets in, the window for intervention is narrow. Early recognition of cutaneous signs and timely detection of anti-MDA5 antibodies are paramount to initiating life-saving immunosuppressive therapy. However, as this case demonstrates, even aggressive treatment can be outpaced by the disease’s relentless progression. The dual threat of respiratory failure and infection from immunosuppression further complicates management, demanding a multidisciplinary approach and patient-centered decision-making. This case underscores the urgent need for targeted therapies and reinforces the message that early intervention may be the only chance to alter the trajectory of this critical illness. 
